# Cyanuric Chloride as an Efficient Catalyst for the Synthesis of 2,3-Unsaturated *O*-Glycosides by Ferrier Rearrangement

**DOI:** 10.1155/2014/307895

**Published:** 2014-01-19

**Authors:** Xiaojuan Yang, Na Li

**Affiliations:** ^1^College of Chemistry and Chemical Engineering, Xinxiang University, Xinxiang, Henan 453003, China; ^2^School of Pharmacy, Xinxiang Medical University, Xinxiang, Henan 453003, China

## Abstract

Cyanuric chloride has been found to be an efficient catalyst for the synthesis of 2,3-unsaturated *O*-glycosides from the reaction of 3,4,6-tri-*O*-acetyl-D-glucal and a wide range of alcohols in dichloromethane at room temperature. The experimental procedure is simple, and the products are obtained in high yields.

## 1. Introduction

2,3-Unsaturated *O*-glycosides play a substantial role in the synthesis of oligosaccharides [1], uronic acids [2], complex carbohydrates [3], and various natural products [4, 5]. Moreover, the unsaturated part of the sugar ring is amenable to easy functionalization such as hydrogenation, hydroxylation, epoxidation, and aminohydroxylation, which contribute to their diversities and complexities. One of the most common procedures to achieve 2,3-unsaturated *O*-glycosides is the acid-catalyzed allyl rearrangement of glucals, which was discovered by Ferrier and Prasad [6]. The Ferrier rearrangement is a reliable procedure for the formation of 2,3-unsaturated-*O*-glycosides, which has known extensive development over decades [7]. This rearrangement typically occurs by treatment of glucals and alcohols with Lewis acids such as BF_3_·Et_2_O [8], FeCl_3_ [9], Montmorillonite K-10 [10], I_2_ [11], InCl_3_ [12], SiO_2_-Bi(OTf)_3_ [13], ZnCl_2_/Al_2_O_3_ [14], or protic acids such as H_2_SO_4_-SiO_2_ [15], H_3_PO_4_ [16], H_2_SO_4_/4 Å molecular sieves [17], CF_3_SO_3_H-SiO_2_ [18], and (*S*)-camphorsulfonic acid [19]; Er(OTf)_3_ [20, 21] as catalyst has also been reported. Although some of these methods have been used for the synthesis of 2,3-unsaturated *O*-glycosides with good to high yields, the majority of them suffer at least from one of disadvantages, such as prolonged reaction times, low yields, harsh reaction conditions, difficulty in the preparation and moisture sensitivity of the catalysts, and high cost and toxicity of the reagents. Therefore, there is a scope to develop an alternative method for the synthesis of 2,3-unsaturated *O*-glycosides.

**Scheme 1:**
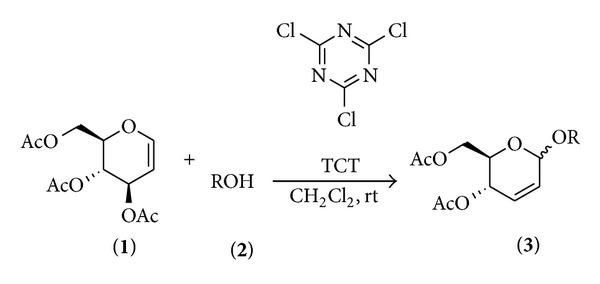
Synthesis of 2,3-unsaturated *O*-glycosides.

Cyanuric chloride (TCT) is an inexpensive, easily available reagent of low toxicity and less corrosiveness than other similar reactants and has been widely used in organic reactions [22–24], but it has not been carefully studied as a catalyst in the synthesis of 2,3-unsaturated *O*-glycosides until now. In this paper, we wish to report a rapid and highly efficient method for the synthesis of 2,3-unsaturated *O*-glycosides in the presence of TCT at room temperature (Scheme 1).

## 2. Experimental

### 2.1. General

^1^H NMR and ^13^C NMR spectra were determined on Bruker AV-400 spectrometer at room temperature using tetramethylsilane (TMS) as an internal standard, coupling constants (*J*) were measured in Hz; elemental analyses were performed by a Vario-III elemental analyzer; commercially available reagents were used throughout without further purification unless otherwise stated.

### 2.2. General Procedure for the Preparation of **3**

To a solution of 3,4,6-tri-*O*-acetyl-D-glucal (272 mg, 1 mmol) in CH_2_Cl_2_ (5 mL) were added the corresponding alcohol (1.2 mmol) and TCT (18 mg, 0.10 mmol). The mixture was stirred at room temperature for an appropriate time (Table 2). After completion of the reaction (TLC), the reaction mixture was treated with 10 mL water and extracted with CH_2_Cl_2_ (3 × 10 mL). The combined organics were dried over anhydrous Na_2_SO_4_. The solvent was removed under vacuum. All the products were purified by silica gel column chromatography (hexane/ethyl acetate = 9/1). The structures of products were identified through comparison of these spectra data with those in the known literatures [8–19]. Spectral data for selected compounds are as follows.

### 2.3. Spectral Data of Some Selected Compounds

*n*-Butyl 4,6-di-*O*-acetyl-2,3-dideoxy-*α*-D-erythro-hex-2-enopyranoside (**3c**). ^1^H NMR (400 MHz, CDCl_3_): **δ** 5.80–5.65 (m, 2H), 5.18 (dd, *J* = 1.2, 9.6 Hz, 1H), 4.90 (s, 1H), 4.06 (dd, *J *= 5.6, 12.4 Hz, 1H), 4.2 (dd, *J* = 2.4, 12 Hz, 1H), 3.98–3.94 (m, 1H), 3.66–3.35 (m, 2H), 1.95 (s, 3H), 1.94 (s, 3H), 1.47–1.23 (m, 4H), 0.81 (t, 3H); ^13^C NMR (100 Hz, CDCl_3_): *δ* 170.5, 170.1, 129.0, 128.1, 93.6, 68.9, 67.0, 65.6, 62.9, 31.8, 20.8, 20.7, 18.9, 13.8. Anal. Calcd. for C_14_H_22_O_6_: C 58.73, H, 7.74. Found: C 58.65; H, 7.69.

Benzyl 4,6-di-*O*-acetyl-2,3-dideoxy-*α*-D-erythro-hex-2-enopyranoside (**3l**). ^1^H NMR (400 MHz, CDCl_3_): **δ**7.33–7.25 (m, 5H), 5.93–5.83 (m, 2H), 5.29 (dd, *J* = 1.6, 2.8 Hz, 1H), 5.12 (s, 1H), 4.82 (d, *J* = 11.6 Hz, 1H), 4.64–4.55 (m, 1H), 4.32–4.08 (m, 3H), 2.07 (s, 3H), 2.05 (s, 3H); ^13^C NMR (100 Hz, CDCl_3_): **δ** 170.5, 170.1, 137.4, 129.5, 128.3, 128.0, 127.8, 127.7, 92.9, 70.6, 67.2, 64.9, 63.0, 20.8, 20.6. Anal. Calcd. for C_17_H_20_O_6_: C 63.74, H, 6.29. Found: C 63.88; H, 6.20.

Cyclohex-2-enyl 4,6-di-*O*-acetyl-2,3-dideoxy-*α*-D-erythro-hex-2-enopyranoside (**3j**). ^1^H NMR (400 MHz, CDCl_3_): **δ**5.87–5.75 (m, 4H), 5.30 (d, *J* = 8.8 Hz, 1H), 5.20 (d, *J* = 18.0 Hz, 1H), 4.27–4.14 (m, 4H), 2.09 (s, 3H), 2.08 (s, 3H), 2.03–1.93 (m, 3H), 1.84–1.70 (m, 2H), 1.56 (m, 1H); ^13^C NMR (100 Hz, CDCl_3_): **δ**170.7, 170.3, 131.6, 128.8, 128.6, 128.4, 128.3, 93.8, 72.9, 67.0, 65.6, 62.9, 29.9, 24.8, 21.2, 19.8. Anal. Calcd. for C_15_H_24_O_6_: C 59.98, H, 8.05. Found: C 60.02; H, 8.02.

Pregnenolonyl 4,6-di-*O*-acetyl-2,3-dideoxy-*α*-D-erythro-hex-2-enopyranoside (**3o**). ^1^H NMR (400 MHz, CDCl_3_): **δ**5.86 (d, *J* = 10.0 Hz, 1H), 5.75 (d, *J* = 10.0 Hz, 1H), 5.31 (s, 1H), 5.24 (d, *J* = 10.0 Hz, 1H), 5.13 (s, 1H), 4.20–3.55 (m, 4H), 2.52 (t, *J* = 8.0 Hz, 1H), 2.38–2.14 (m, 3H), 2.11 (s, 3H), 2.06 (s, 3H), 2.05 (s, 3H), 2.02 (d, *J* = 9.5 Hz, 1H), 1.99–1.42 (m, 11H), 1.23–1.11 (m, 2H), 1.06–0.96 (m, 5H), 0.62 (s, 3H); ^13^C NMR (100 Hz, CDCl_3_): **δ**208.9, 170.8, 170.3, 140.5, 128.7, 128.2, 121.3, 93.0, 78.2, 67.0, 65.5, 63.7, 63.2, 56.4, 50.0, 44.1, 40.5, 38.3, 37.3, 36.2, 31.8, 31.7, 31.4, 27.9, 24.3, 22.8, 21.2, 21.0, 20.8, 19.5, 13.5. Anal. Calcd. for C_31_H_44_O_7_: C 70.43, H, 8.39. Found: C 70.29; H, 8.27.

## 3. Results and Discussion

In a typical reaction procedure, 2,3-unsaturated *O*-glycosides **3** were prepared, from the reaction of 3,4,6-tri-*O*-acetyl-D-glucal **1** and alcohols **2** in CH_2_Cl_2_ at room temperature in the presence of TCT as catalyst (Scheme 1). Since in this new kind of methodology, there is a great challenge to find new catalysts able to perform the reactions with high activities, we considered TCT as a potential promoter due to its good features for such a transformation. During our investigation, we firstly chose the reaction of 3,4,6-tri-*O*-acetyl-D-glucal (1 mmol) and methanol (1.2 mmol) as model reactants and examined the effect of the different amounts of TCT (Table 1). When above model reactants were conducted in CH_2_Cl_2_ (5 mL) in the presence of 0, 0.05, 0.10, 0.15, and 0.20 mmol of TCT separately, the best result was obtained using 0.10 mmol of catalyst (yield = 90%). Using lower amounts of catalyst resulted in lower yields, while higher amounts of catalyst did not affect the reaction times and yields (Table 1, entries 2–5). In the absence of catalyst, the product was not formed (Table 1, entry 1). In order to evaluate the effect of the solvent, the reaction of 3,4,6-tri-*O*-acetyl-D-glucal and methanol in the presence of TCT was performed in various solvents such as CH_2_Cl_2_, CH_3_CN, acetone, THF, and ethyl ether, in which the best result was obtained in CH_2_Cl_2_ (Table 1, entry 3); thus, we chose it as the solvent for all further reactions (Table 1, entries 6–9).

Under the optimum conditions, a wide range of alcohols, including primary, secondary, and allylic alcohols, reacted with acetylated glucal according to Scheme 1 using 10 mol % of TCT at room temperature to give the corresponding 2,3-unsaturated *O*-glycosides in good to excellent yields and in very short reaction time with *α*-anomer as a major product (Table 2).

Encouraged by these results, we next explored the scope of this methodology for the synthesis of pseudoglycals connected to various biologically important natural products (Table 2, entries 15–17). Under our catalytic conditions, pregnenolone, L-menthol, and borneol pseudoglycosides were obtained in 86%, 88%, and 85% yields, respectively, with exclusive *α*-anomeric selectivity.

**Scheme 2:**
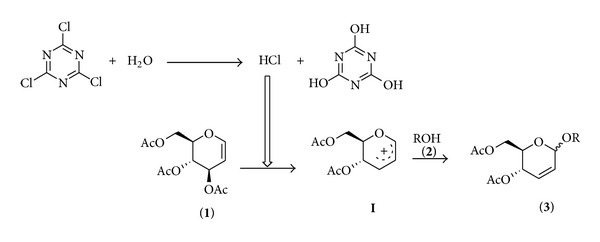
Plausible mechanism for the formation of 2,3-unsaturated *O*-glycosides.

The formation of products can be explained as follows: initially TCT reacts with the adventitious moisture to form 3 mol of HCl and cyanuric acid (removable by water washing) as a byproduct. The *in situ* generated HCl assists the formation of the intermediate cyclic allylic oxocarbenium ion **I** from 3,4,6-tri-*O*-acetyl-D-glucal, which was originally proposed by Ferrier. Then, alcohol is added to the intermediate **I** in a quasiaxial fashion preferentially to provide the product 2,3-unsaturated *O*-glycosides **3** having major *α*-selectivity as shown in Scheme 2.

## 4. Conclusion

In conclusion, we have developed a mild, eco-friendly approach for the synthesis of 2,3-unsaturated *O*-glycosides using a catalytic quantity of TCT. The main advantages of this method include good *α*-selectivity, the use of inexpensive catalyst, and environmentally benign character. We believe that the methodology reported here is synthetically quite attractive and would spur on further interest toward the synthesis of complex glycosides.

## Figures and Tables

**Table 1:** Reaction conditions optimisation for the synthesis of **3a**.

Entry	Catalyst/mmol	Solvent	Time/min	Yield/%
1	0	CH_2_Cl_2_	120	Trace
2	0.05	CH_2_Cl_2_	60	79
3	0.10	CH_2_Cl_2_	30	90
4	0.15	CH_2_Cl_2_	30	89
5	0.20	CH_2_Cl_2_	30	90
6	0.10	CH_3_CN	30	82
7	0.10	Acetone	30	80
8	0.10	THF	30	69
9	0.10	Ethyl ether	60	42

**Table 2:** Preparation of 2,3-unsaturated *O*-glycosides.

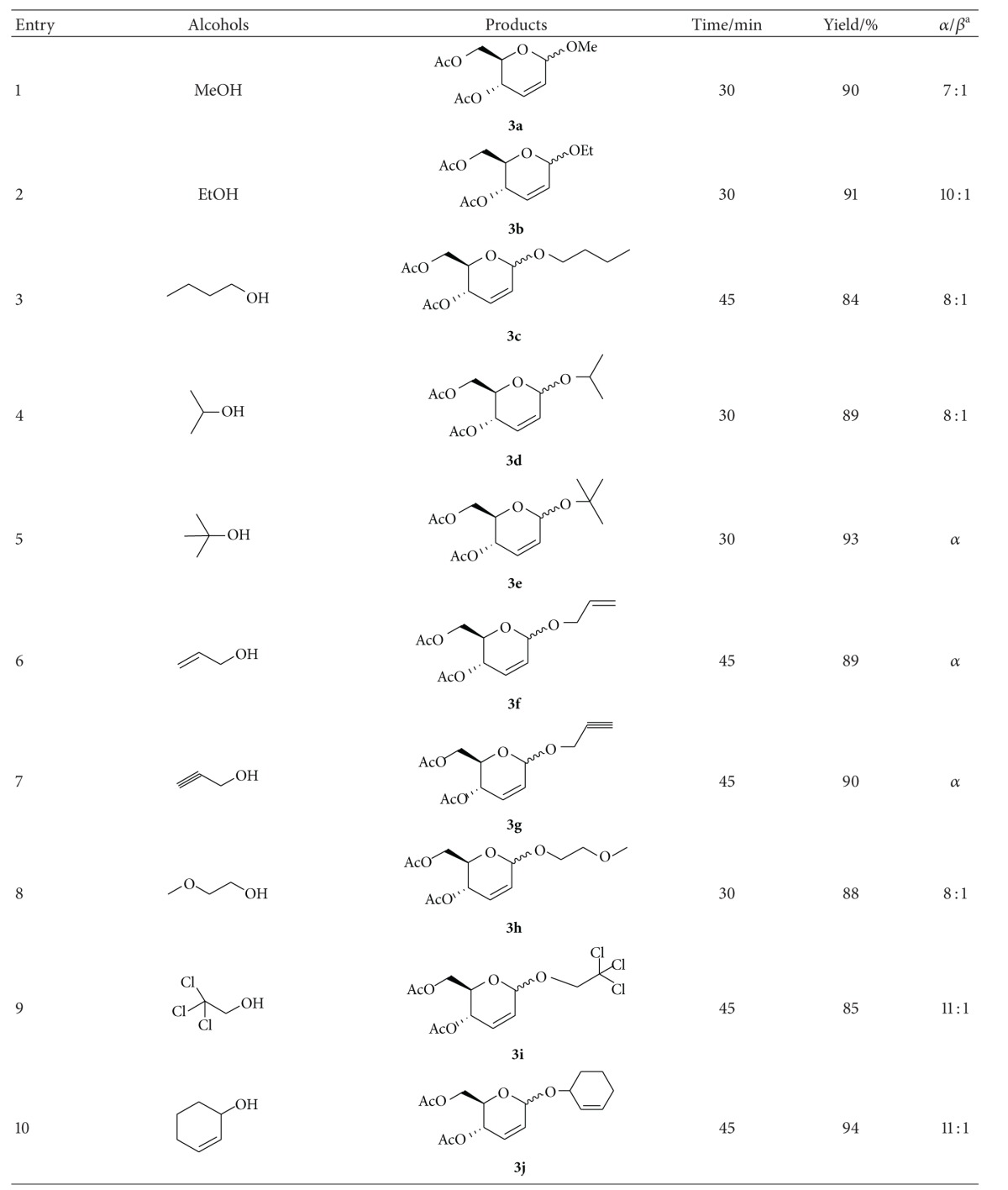 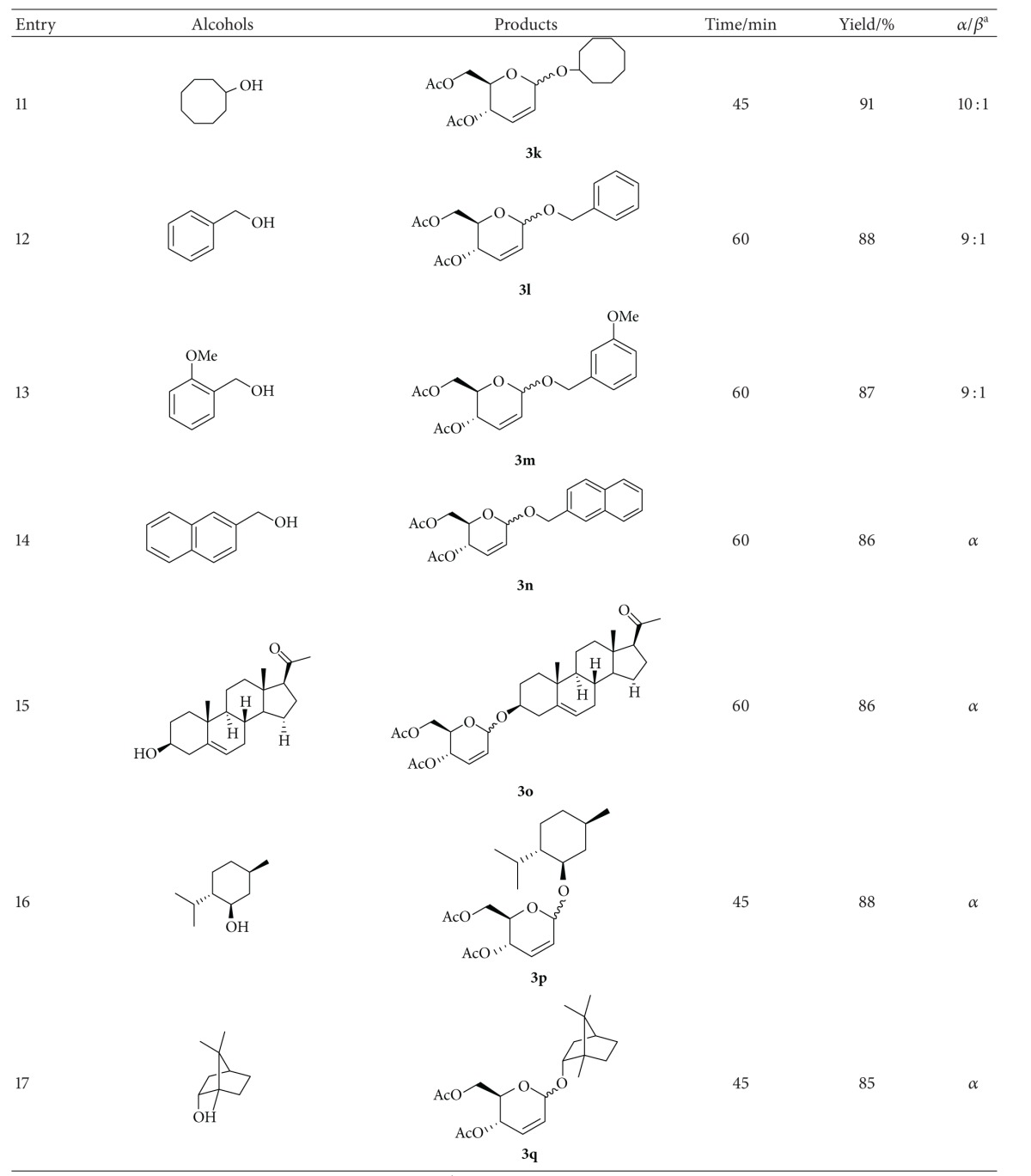

^a^The *α*/*β* ratio was determined from the anomeric proton ratio in the ^1^H NMR spectra.
